# Noncoding RNA in the Regulation of Acute Aortic Dissection: From Profile to Mechanism

**DOI:** 10.1155/2022/2371401

**Published:** 2022-11-18

**Authors:** Ruibin Wei, Yingqing Feng

**Affiliations:** ^1^Department of Cardiology, Panyu Central Hospital, East Fuyu Road, Qiaonan Street, Panyu District, Guangzhou, China; ^2^The Second School of Clinical Medicine, Southern Medical University, Guangzhou, China; ^3^Department of Cardiology, Guangdong Cardiovascular Institute, Affiliated South China Hospital, Southern Medical University (Guangdong Provincial People's Hospital) Guangdong Academy of Medical Sciences, Guangzhou, China

## Abstract

Aortic dissection is a life-threatening condition caused by a tear in the intimal layer of the aorta or bleeding within the aortic wall, resulting in the separation of the layers of the aortic wall. As Nienaber reported, aortic dissection is most common in people 65–75 years old and has an incidence of 35 cases per 100,000 people per year in this population. Many pathogenic factors are involved in aortic dissection, including hypertension, dyslipidemia, and abnormality of the aortic intima caused by genetic variation. However, with the development of gene sequencing and transgenic technology, genetic methods are being used for the diagnosis and treatment of diseases, including acute aortic dissection. Genetic research on acute aortic dissection began around 2006. Recently, research on acute aortic dissection has mainly focused on microRNA (miRNA). Studies have found that miRNA plays a critical regulatory role in the occurrence and development of acute aortic dissection. By regulating miRNA expression, acute aortic dissection can be prevented and treated.

## 1. miRNAs and Aortic Dissection

miRNAs are small single-stranded noncoding RNA molecules containing about 22 nucleotides found in plants, animals, and some viruses that function in RNA silencing and the posttranscriptional regulation of gene expression [1]. Because of their conservative sequences and the small number of bases, scientists have studied miRNAs as cardiovascular disease markers [2] and therapeutic drug targets [3]. However, there are few studies on miRNA and acute aortic dissection, and the research methods are relatively simple, which hinders the development of miRNA markers and therapeutic drugs for aortic dissection.

There are two main methods to study aortic dissection and miRNA: first, extract miRNA from patient aortic tissue and compare it with normal aortic tissue to obtain differentially expressed miRNAs and then test stimulators or inhibitors of these miRNAs in animals, which is currently the mainstream method. The other is to extract the plasma of patients with aortic dissection and analyze the miRNA expression profile compared to healthy people to identify differentially expressed miRNA. This research method is less often used and lacks experimental animal verification. The former research method is less affected by confounding factors, and the former research thinking is meticulous. In contrast, the latter research method is more macroscopic and holistic, for its research source is from circulating blood, and its clinical value is higher than the former to a certain extent. Overall, there are advantages and disadvantages of each of the above two approaches, and finding new research methods that balance the advantages and disadvantages of these approaches will help researchers gain a deeper understanding of the pathogenesis of miRNA and aortic dissection.

Research on miRNA and aortic dissection began in 2011. Liao et al. first extracted tissue from the aorta of patients with thoracic aortic dissection, sequenced their DNA, and compared it with normal aortic tissue. They found that 18 miRNAs were upregulated and 56 were downregulated (fold change > 2, *P* < 0.01). They then confirmed by qRT-PCR that seven of the screened miRNAs were statistically consistent. That study also explored the downstream pathway of these miRNAs and found them to be related to cell adhesion and the MAPK pathway, which paved the way for later exploration of the underlying mechanism [4]. Wang et al. also found 30 abnormally expressed miRNAs in aortic tissue of patients with acute aortic dissection, including 13 upregulated and 17 downregulated miRNAs, using high-throughput sequencing. Additional research revealed that the downstream genes of these miRNAs are related to many pathways such as intercellular adhesion, extracellular matrix metabolism, inflammation, and the cell cycle [5]. Neither of these studies explored the underlying mechanism but only described the correlation.

Research on the regulatory mechanism of miRNA in patients with aortic dissection began in 2017. However, it is not a simple study of aortic dissection but also involves aortic aneurysms. Yu et al. extracted aortic tissue from aortic dissection and aortic aneurysm patients. They found that *miR-30a* was significantly overexpressed, while lysyl oxidase (*LOX*) and elastin were significantly low-expressed in these patients. Later, after luciferase detection, it was found that *miR-30a* could specifically bind to *LOX* and regulate the expression of *LOX* in vascular smooth muscle cells (VSMCs). Finally, animal experiments further confirmed that the inhibition of miR-30a expression inhibited the occurrence of aortic dissection in mice [6]. This was the first study on target miRNA and aortic dissection found in humans that was validated in animals. Nearly all of the subsequent articles followed this method to explore the relationship between miRNA and aortic dissection, involving changes in cell phenotype or the regulation of signaling pathways, which further revealed the regulatory mechanisms of miRNA. For example, Sun et al. found that *miR-27a* regulates vascular remodeling via endothelial cell apoptosis and interactions with VSMCs. Additional studies in mice found that overexpressed *miR-27a* inhibits the occurrence of aortic dissection [7]. Wang et al. demonstrated that *miR-134-5p* inhibits the phenotypic transformation and migration of VSMCs by targeting the gene *STAT5B/ITGB1*, thus regulating aortic dilatation and vascular medial degeneration. In mice where *miR-134-5p* was overexpressed, thoracic aorta dilation is inhibited, and medial vascular degeneration is reduced by 39% [8].

Additionally, studying the relationship between miRNA and dissection as an upstream factor can be used as an intermediate factor to prevent aortic dissection. For example, Duan et al. found that *miR-133a* is upregulated by adiponectin, thus inhibiting the pyroptosis pathway of cells, and a series of inflammatory factors such as caspase-1, interleukin-1 *β* (IL-1 *β*), IL-18, and osteopontin (OPN) are inhibited, resulting in aortic dissection [9].

Studies on the differential expression of miRNA in patients with aortic dissection are still in progress. The differentially expressed and regulated miRNAs detected thus far in aortic tissues do not have the same differential expression in the plasma of these patients, or the authors of studies have not mentioned this aspect at all. Therefore, we believe that studying aortic dissection, a circulatory disease, solely from miRNAs with differential tissue expression does not provide a comprehensive picture of the nature of the disease. Additionally, aortic dissection must occur due to the abnormal expression of circulating miRNAs or other genes that cause the differential expression of miRNAs in aortic tissues and thus affect the disease process.

As mentioned above, the miRNA extracted from the aorta is less affected. However, whether these miRNAs still play a role in complex intraorganismal environments needs further exploration. On the contrary, although miRNAs in human circulation are affected by many factors, if they are validated to regulate aortic dissection, their clinical significance is higher than that of miRNAs in tissue.

Research on the relationship between circulating miRNA and aortic dissection began in 2015. As mentioned above, Wang et al. identified 30 abnormally expressed miRNAs in patient aorta tissue, and there was a significant difference in the expression of miRNA in plasma. Twenty miRNAs (10 upregulated and 10 downregulated) were expressed more than 20× that in healthy controls, and four miRNAs were significantly differentially expressed in patients' plasma and tissues compared with normal controls. However, the difference in plasma expression was significantly higher than that in tissues, most of which were more than ten times the expression seen in tissues [5]. In that study, miRNA expression in circulation was inconsistent with that in tissue. However, whether miRNA regulation in tissue or circulation plays a dominant role in the occurrence and development of aortic dissection requires more research.

In another study on circulating miRNA and aortic dissection, Wang et al. screened 21 miRNAs from the plasma of 98 patients, among which there was a significant difference between *miR-4787-5p* and *miR-4306*. Moreover, another study demonstrated that both miRNAs had high specificity and sensitivity in the diagnosis of aortic dissection, in which the area under the receiver operating characteristic (AUC) curve of *miR-4787-5p* was 0.898 (95% confidence interval [CI], 0.847–0.948). The area under the ROC curve of *miR-4306* was 0.874 (95% CI, 0.820–0.927). When the two were evaluated together, the area under the ROC curve was as high as 0.961 (95% CI, 0.820–0.927), which is higher than that of D-dimer [10]. Meanwhile, that paper also investigated downstream targets of these miRNAs and their possible regulation. However, these two miRNAs were not further verified in mice, so the specific mechanism of their regulation in aortic dissection needs to be further researched.

Dong et al. published an article in 2017 specifically studying plasma miRNA as a marker of aortic dissection. In this paper, the target miRNA was identified in three groups of patients and then further verified. *miR-15a* could distinguish patients with acute fatal chest pain and effectively differentiate between healthy people and those with acute myocardial infarction, aortic aneurysm, or pulmonary embolism. Its expression level was significantly increased in patients with acute aortic dissection. Additionally, *miR-23a* is effective in distinguishing those with acute aortic dissection from those with chest pain diseases, with a sensitivity of 75.7% and a specificity of 100% [11]. However, as in Wang et al., there was no mechanistic research, no verification in animal experiments, and the number of patients included was small. Therefore, its accuracy is still open to question.

miRNA in tissue is related to active surface dissection, but whether there is such a relationship with circular miRNA has not been confirmed. Moreover, in the three studies mentioned above, the difference in miRNA expression in the plasma of the same patients with aortic dissection was different; whether there are differences in various populations also needs to be explored in additional experiments.

Research into miRNA and aortic dissection has been ongoing for some time. However, a future research direction will no longer be to screen for differentially expressed miRNAs but to explore miRNAs that share differential expression in tissues and circulation from an integrative perspective, which will address the advantages and disadvantages mentioned above and provide more compelling miRNA targets for drug therapy in aortic dissection. However, there may be a long road ahead [12].

## 2. Long Noncoding RNAs and Aortic Dissection

Long noncoding RNA (lncRNA) is a class of noncoding RNAs greater than 200 nucleotides in length. These are now recognized as playing crucial roles in numerous cellular processes, including the cell cycle, differentiation, and metabolism as well as in disease [13].

Li et al. studied patients with thoracic aortic dissection [14]. Aortic tissue specimens from patients were extracted, and 765 lncRNAs and 619 genes with differential mRNA expression were identified by sequencing (fold change > 2.0, *P* < 0.01). They then performed gene ontology (GO) analysis and found that lncRNAs with upregulated expression were associated with cell differentiation, homeostasis, growth, and proliferation. KEGG analysis of downregulated lncRNAs showed that they were associated with arrhythmogenic right ventricular cardiomyopathy, hypertrophic cardiomyopathy, and dilated cardiomyopathy. Increasing the fold change narrowed the number to 16 lncRNAs, and they found that these lncRNAs were associated with protein-coding genes. That study then confirmed by RT-qPCR that the lncRNAs *P2RX7*, *(HIF)-1A-AS2*, *AX746823*, *RP11-69I8.3,* and *RP11-536 K7.5* were associated with P2RX7, cyclin-dependent kinase inhibitor 2B, HIF-1A, runt-related transcription factor 1, connective tissue growth factor, and interleukin 2 receptor chain mRNAs. In contrast, these lncRNAs were associated with nuclear receptor activation, nuclear transcription, connective tissue development, and inflammation.

Sun et al. also examined lncRNA expression profiles in tissues from patients with aortic dissection. Unlike Li et al., Sun et al. used high-throughput sequencing, which yielded 269 lncRNAs and 2255 mRNAs. lncRNA-miRNA-mRNA network analysis revealed that both the upregulated lncRNA *XIST* and p21 had similar sequences of *miR-17-5p*. Additionally, the predicted binding motifs of three upregulated lncRNAs (ENSG00000248508, ENSG00000226530, and EG00000259719) were associated with upregulated RUNX1 [15].

The above two studies on lncRNAs and aortic dissection initially revealed differentially expressed lncRNAs in the tissues of patients with thoracic aortic dissection and explored the relationship between significantly differentially expressed lncRNAs and their related downstream mRNAs, paving the way for later studies. Although the sequencing methods used in the two articles were different, neither report investigated related mechanisms, only their expression profiles. The pathways were not explored, and the cellular phenotypic changes were not investigated in depth. Therefore, additional studies were needed to study the relationship between lncRNAs and aortic dissection.

To address this, Zhang et al. analyzed the aortic tissues of Stanford type A dissection patients and identified the lncRNA *XIST*. They investigated its molecular mechanism by dual luciferase reporters, qPCR, and Western blot experiments. They found that *XIST* regulates the expression of PTEN through the sponge *miR-17*, which affects the proliferation and regulation of VSMCs. Overexpression of *XIST* promotes apoptosis and inhibits the proliferation of VSMCs, which may lead to the development of aortic dissection [16]. Immediately after, Li et al. showed that downregulation of the lncRNA *PVT1* inhibited the survival, migration and phenotypic transition of platelet-derived growth factor-BB (PDGF-BB) treated human aortic smooth muscle cells by targeting *miR-27b-3p* [17]. Ren et al. found that overexpression of the lncRNA *H19* sponged *miR-193b-3p* to regulate VSMC function, including upregulating its proliferation and migration, while these effects appeared to be reversed after inhibiting *H19* expression. Thus, they concluded that *H19* may be involved in the development of aortic dissection [18]. Wang et al. showed that *LINC01278* regulated the expression of the *ACTG2* gene through the sponge *miR-500b-5p*, regulating the phenotypic transformation, proliferation, and migration of human VSMCs [19].

Changes in VSMCs are an important mechanism of aortic dissection. However, the tunica media becomes fragile in aortic dissection. Additionally, damage is closely related to changes in elastin and collagen, whose degradation was accounted for the family of matrix metalloproteinases (MMPs). Xu et al. extracted lncRNAs from the tissues of patients with type B dissection and found 393 abnormally expressed lncRNAs and 432 abnormally expressed mRNAs. Among them, *TNFSF14* was negatively correlated with *MMP14* and *MMP19* (the Pearson correlation coefficient *r*_s_ was between -7.0 and 8.5). Finally, *lnc-TNSF14* may play a key role in regulating matrix degradation, which may affect the development of type B aortic dissection [20].

Bioinformatics analysis has also been used to study lncRNAs and aortic dissection, especially to identify lncRNA-miRNA-mRNA networks, providing valuable information for verifying underlying mechanisms. Shao et al. analyzed abnormally expressed lncRNAs, miRNAs, and mRNAs in thoracic aortic dissection using the GEO database; analyzed differentially expressed mRNAs by KEGG and GO analyses; constructed a protein-protein interaction network of differentially expressed mRNAs; identified hub genes. Finally, their results were verified in another database [21]. Similarly, Zhang et al. found three GEO series (GSEs) in the GEO database, with each RNA corresponding to a GSE, and then they selected three lncRNAs, five miRNAs, and 211 mRNAs. Meanwhile, they analyzed the genes in the constructed ncRNA network and identified four pathway axes: *lncRNA-FAM87A-has-miR-31-5p/has-miR-7-5p-E2F2*, *lncRNA-C9orf106-hsa-miR-7-5p-E2F2*, *lncRNA-FAM87A-hsa-miR-16-5p/hsa-miR-7-5p-IGF1R,* and *lncRNA-C9orf106-hsamiR-7-5p-IGF1R* [22]. These four pathway networks help us understand possible pathogenic mechanisms of aortic dissection and provide potential critical targets for its treatment.

Research on lncRNAs and aortic dissection has mainly been carried out only recently. Because of the tissue specificity lncRNAs, no relationship between plasma lncRNAs and aortic dissection has been detected. For this reason, scientists have not conducted animal experiments to further verify the mechanism of lncRNAs and aortic dissection in tissues. lncRNA may play a critical role in regulating the development of aortic dissection. However, because aortic dissection is a disease involving both plasma and tissue, lncRNA may be only an intermediate molecule in the disease mechanism, acting as an enabler and not as an initiator of aortic dissection.

## 3. circRNAs and Aortic Dissection

circRNAs are recently discovered regulatory molecules with stable properties. Most circRNAs compete for endogenous RNAs (ncRNAs) and show tissue and developmental stage-specific expression [23]. Moreover, circRNAs may be involved in vascular function. For example, 4464 tissue-specific circRNAs were detected in the normal human aorta [24]. Also, a recent study showed that circRNAs were aberrantly expressed in hypoxia in human umbilical endothelial cells and exhibited a physiological function *in vitro* [25]. Furthermore, the circRNA *cANRIL* may be correlated with atherosclerotic vascular disease risk *in vitro* [26, 27].

Research on the relationship between circRNAs and aortic dissection began in 2017. Like lncRNA, the study of circRNA and aortic dissection also begins with sequencing aortic tissue. Zou et al. first sequenced aorta tissues from patients with aortic dissection and found 8173 expressed circRNAs, of which 156 were upregulated (fold change ≥ 1.5) and 106 were downregulated. Then, GO analysis showed that the upregulated circRNAs were related to cell proliferation and the regulation of the extracellular matrix. The downregulated circRNA was related to actin structure and the actin cytoskeleton. From these circRNAs, 33 with at least one target miRNA were selected. Among these, *circRNA_101238* was associated with three miRNAs. Finally, through qPCR and double luciferase reporter experiments, they showed that *circRNA_101238* can adsorb *miR-320a* to regulate the activity of MMP9, which may affect the occurrence and development of aortic dissection [28].

Thus far, there is only one study on the mechanism of circRNA and aortic dissection. However, this study was rigorous and involved basic research on circRNAs. In Xu et al., *circ_TGFBR2* was selected from previous studies and was then verified in aortic dissection and normal aortic tissue. That study confirmed the significantly low expression of *circ_TGFBR2* in aortic dissection tissue and VSMCs compared to healthy tissues. Second, its ring structure was verified by RNase R, and the expression of *circ_TGFBR2* in the cytoplasm was confirmed by *in situ* hybridization. Third, by downregulating the expression of *circ_TGFBR2* in normal aortic VSMCs, the proliferation, migration, and phenotypic transition of VSMCs were promoted; by upregulating the expression of *circ_TGFBR2* in VSMCs from aortic dissection patients, the proliferation, migration, and phenotypic transition of VSMCs were inhibited. Furthermore, the high binding of *miR-29a* and wild-type *circ_TGFBR2* was confirmed by luciferase reporter experiments, and these results were confirmed again by an RNA pulldown test. Finally, a rescue test confirmed that lentivirus cotransfection with *miR-29a* reversed the effect of *circ_TGFBR2* in VSMCs from aortic dissection patients. Additionally, to further confirm the mechanism of *miR-29a* in VSMCs, the downstream target gene *KLF4* of *miR-29a* was identified by bioinformatics analysis. A luciferase reporter experiment demonstrated that *miR-29a* and wild-type *KLF4* expression was negatively correlated. In VSMCs from aortic dissection patients, *KLF4* inhibited the expression of proliferation, migration, and synthetic markers and increased the expression of apoptosis and contraction markers. A rescue experiment confirmed that the effect of *KLF4* could be rescued by *miR-29a*. It is worth mentioning that these studies were performed in mice. It was confirmed by lentivirus transfection of *circ_TGFBR2* that it can sponge *miR3-29a* to regulate the proliferation, migration, and phenotypic transformation of VSMCs through KLF4, affecting the morphology of the aorta and the occurrence and development of dissection. These results provide a novel circRNA target for future studies of markers and drugs [29].

In a study by Xu et al., *circ_TGFBR2* was found to play a pivotal role in patients with aortic dissection, regulating VSMC phenotypic changes in aortic tissue and the circulation of rats. We believe this model will be a template for future studies of aortic dissection and provides a research direction to reveal the molecular mechanisms of aortic dissection.

Additionally, the superior stability of circRNAs allowed them to be used as a biomarker for aortic dissection with a specificity of 86.7% and sensitivity of 90%. Tian et al. found that *circMARK3* as a marker had a high diagnostic value for the diagnosis of Stanford type A aortic dissection (cutoff value = 1.497, area under the curve = 0.9344, *P* < 0.0001, sensitivity = 90.0%, specificity = 86.7%) [30].

Unlike lncRNAs, there are few studies on the role of circRNAs in aortic dissection. However, the article by Xu et al. is mechanistically rigorous and involves animal experiments, which is lacking in many studies on lncRNAs and aortic dissection. Although studies of lncRNAs and aortic dissection also involve signaling pathways, their rigor is weakened by the tissue-specific expression of lncRNAs, which made it impossible to validate these pathways in animals. In contrast, circRNAs and aortic dissection, circRNAs and its sponge miRNAs and the miRNA-regulated downstream genes have been comprehensively and systematically experimentally validated. Future research on aortic dissection should focus on circRNAs.

## 4. Discussion

As mentioned above, ncRNAs and aortic dissection studies mainly include miRNAs, lncRNAs, and circRNAs. These studies, without exception, investigate profiles to mechanisms. This is a gradually developing process, and the relationship between molecules or the mechanism underlying the molecular regulation of aortic dissection is discussed. Additionally, animal experiments have been carried out to further confirm these findings.

In profiling studies, many experiments have used high-throughput sequencing to extract many ncRNAs from aortic tissue or patient blood. Then, bioinformatics analysis is used to obtain expression networks of downstream genes, which provides valuable information for future research. However, ncRNAs have only been extracted from tissue or plasma, but not both. Aortic dissection is a disease of both tissue and blood; aortic tissue is the internal cause and blood is the external cause. The occurrence of aortic dissection is inseparable from the joint action of internal and external factors. Unfortunately, only one study on circRNAs in aortic dissection involved both tissue and blood. In contrast, other studies only explored one or the other without integrating both factors, which is unlikely to solve clinical problems. Moreover, research on the regulatory role of ncRNAs in aortic dissection focuses on the effect of miRNAs or lncRNAs on VSMCs. Subsequent research needs to be extended to include other cellular components of blood vessels, such as endothelial cells, fibroblasts, and macrophages [31].

In mechanistic studies, miRNA is an important bridge connecting the two types of ncRNAs, lncRNA, and circRNA, with regulatory genes. miRNA also plays a role in regulating the occurrence and development of aortic dissection through gene regulation. However, miRNA and lncRNA studies have not involved the same molecules coexpressed in plasma and tissues, so further exploration is still needed.

In conclusion, both profiling and mechanistic studies on ncRNA have found that it is closely related to the occurrence and development of aortic dissection. However, due to the complexity of aortic dissection, no ncRNAs have been found as biomarkers of aortic dissection, and no effective drug therapy targets for this disease have been found. These two aspects will be the direction and focus of future research.

## Data Availability

The data supporting this review are from previously reported studies and datasets, which have been cited.

## References

[1] Bartel D. P. (2018). Metazoan microRNAs. *Cell*.

[2] Wang B., Li Y., Hao X. (2021). Comparison of the clinical value of miRNAs and conventional biomarkers in AMI: a systematic review. *Frontiers in Genetics*.

[3] Täubel J., Hauke W., Rump S. (2021). Novel antisense therapy targeting microRNA-132 in patients with heart failure: results of a first-in-human phase 1b randomized, double-blind, placebo-controlled study. *European Heart Journal*.

[4] Liao M., Zou S., Weng J. (2011). A microRNA profile comparison between thoracic aortic dissection and normal thoracic aorta indicates the potential role of microRNAs in contributing to thoracic aortic dissection pathogenesis. *Journal of Vascular Surgery*.

[5] Wang X. J., Huang B., Yang Y. M. (2015). Differential expression of microRNAs in aortic tissue and plasma in patients with acute aortic dissection. *Journal of Geriatric Cardiology*.

[6] Yu Y., Shi E., Gu T. (2017). Overexpression of microRNA-30a contributes to the development of aortic dissection by targeting lysyl oxidase. *The Journal of Thoracic and Cardiovascular Surgery*.

[7] Sun Y., Xiao Y., Sun H. (2019). miR-27a regulates vascular remodeling by targeting endothelial cells’ apoptosis and interaction with vascular smooth muscle cells in aortic dissection. *Theranostics*.

[8] Wang Y., Dong C. Q., Peng G. Y. (2019). MicroRNA-134-5p regulates media degeneration through inhibiting VSMC phenotypic switch and migration in thoracic aortic dissection. *Molecular Therapy-Nucleic Acids*.

[9] Duan H., Zhang X., Song R., Liu T., Zhang Y., Yu A. (2020). Upregulation of miR-133a by adiponectin inhibits pyroptosis pathway and rescues acute aortic dissection. *Acta Biochimica et Biophysica Sinica*.

[10] Wang L., Zhang S., Xu Z., Zhang J., Li L., Zhao G. (2017). The diagnostic value of microRNA-4787-5p and microRNA-4306 in patients with acute aortic dissection. *American Journal of Translational Research*.

[11] Dong J., Bao J., Feng R. (2017). Circulating microRNAs: a novel potential biomarker for diagnosing acute aortic dissection. *Scientific Reports*.

[12] Sbarouni E., Georgiadou P. (2018). MicroRNAs in acute aortic dissection. *Journal of Thoracic Disease*.

[13] Bridges M. C., Daulagala A. C., Kourtidis A. (2021). LNCcation: lncRNA localization and function. *The Journal of Cell Biology*.

[14] Li Y., Yang N., Zhou X. (2018). lncRNA and mRNA interaction study based on transcriptome profiles reveals potential core genes in the pathogenesis of human thoracic aortic dissection. *Molecular Medicine Reports*.

[15] Sun J., Chen G., Jing Y. (2018). lncRNA expression profile of human thoracic aortic dissection by high-throughput sequencing. *Cellular Physiology and Biochemistry*.

[16] Zhang X., Wu H., Mai C., Qi Y. (2020). Long noncoding RNA XIST/miR-17/PTEN Axis modulates the proliferation and apoptosis of vascular smooth muscle cells to affect Stanford type a aortic dissection. *Journal of Cardiovascular Pharmacology*.

[17] Li S., Zhao X., Cheng S., Li J., Bai X., Meng X. (2021). Downregulating long non-coding RNA PVT1 expression inhibited the viability, migration and phenotypic switch of PDGF-BB-treated human aortic smooth muscle cells via targeting miR-27b-3p. *Human Cell*.

[18] Ren M., Wang T., Wei X. (2021). lncRNA H19 regulates smooth muscle cell functions and participates in the development of aortic dissection through sponging miR-193b-3p. *Bioscience Reports*.

[19] Wang W., Liu Q., Wang Y. (2021). LINC01278 sponges miR-500b-5p to regulate the expression of ACTG2 to control phenotypic switching in human vascular smooth muscle cells during aortic dissection. *Journal of the American Heart Association*.

[20] Xu H., Zhang B., Li Y. (2021). Dysregulated long non-coding RNAs involved in regulation of matrix degradation during type-B aortic dissection pathogenesis. *General Thoracic and Cardiovascular Surgery*.

[21] Shao Y., Luo J., Ye L. (2021). Construction and integrated analysis of competitive endogenous long non-coding RNA network in thoracic aortic dissection. *International Journal of General Medicine*.

[22] Zhang H., Bian C., Tu S. (2021). Integrated analysis of lncRNA-miRNA-mRNA ceRNA network in human aortic dissection. *BMC Genomics*.

[23] Guo J. U., Agarwal V., Guo H., Bartel D. P. (2014). Expanded identification and characterization of mammalian circular RNAs. *Genome Biology*.

[24] Xia S., Feng J., Lei L. (2017). Comprehensive characterization of tissue-specific circular RNAs in the human and mouse genomes. *Briefings in Bioinformatics*.

[25] Boeckel J. N., Jaé N., Heumüller A. W. (2015). Identification and characterization of hypoxia-regulated endothelial circular RNA. *Circulation Research*.

[26] Burd C. E., Jeck W. R., Liu Y., Sanoff H. K., Wang Z., Sharpless N. E. (2010). Expression of linear and novel circular forms of an INK4/ARF-associated non-coding RNA correlates with atherosclerosis risk. *PLoS Genetics*.

[27] Holdt L. M., Stahringer A., Sass K. (2016). Circular non-coding RNA *ANRIL* modulates ribosomal RNA maturation and atherosclerosis in humans. *Nature Communications*.

[28] Zou M., Huang C., Li X. (2017). Circular RNA expression profile and potential function of hsa_circRNA_101238 in human thoracic aortic dissection. *Oncotarget*.

[29] Xu Z., Zhong K., Guo G. (2021). circ_TGFBR2 inhibits vascular smooth muscle cells phenotypic switch and suppresses aortic dissection progression by sponging miR-29a. *Journal of Inflammation Research*.

[30] Tian C., Tang X., Zhu X. (2019). Expression profiles of circRNAs and the potential diagnostic value of serum circMARK3 in human acute Stanford type A aortic dissection. *PLoS One*.

[31] Hu Y.-Y., Cheng X.-M., Wu N., Tao Y., Wang X.-N. (2022). Non-coding RNAs regulate the pathogenesis of aortic dissection. *Frontiers in Cardiovascular Medicine*.

